# King Edward's Hospital Fund

**Published:** 1907-06-08

**Authors:** 


### KING EDWARD'S HOSPITAL FUND.

The *Times* reports that the Bill for the incorporation of King Edward's Hospital Fund for London, which has passed through all its stages in the House of Lords, was considered on the 30th ult. by the Select Committee of the House of Commons on Unopposed Bills, Mr. Emmott in the chair.

Mr. Cameron, Parliamentary agent, stated that the Bill emanated from his Royal Highness the Prince of Wales, with the express approval and sanction of his Majesty the King. The object of the Bill was to incorporate the president, who was the Prince of Wales, and General Council of the Hospital Fund, and to provide for the management of the fund. The General Council acted as an advisory committee to the president, but all the money was vested in the Prince of Wales, who had absolute control of the funds.

In reply to Sir Chandos Leigh, the Speaker's counsel, Mr. Cameron, said the object sought to be attained by the Bill could not have been achieved by a charter, and, moreover, the King had a decided objection to a charter, as his Majesty did not think that was a proper method of bringing the matter forward. Replying to questions by the committee, Mr. Cameron stated that the subscribers had not been called together and consulted with regard to the proposed incorporation—that was an impossibility, having regard to the number of subscribers—but the Bill had been submitted to the council and unanimously approved by them. What the Bill did was to give statutory form to the method of administration which had existed since the fund was established in 1897.

Mr. Caldwell (of the committee) took strong exception to the whole management of the fund, which now amounted to over £1,000,000, being given to one person; and when Mr. Cameron replied that this was a case of the administration of a trust which had been confided to the Prince of Wales, the Chairman pointed out that the Bill gave the same power to future presidents, Mr. Beale (of the committee) adding that the president was irremovable.

It was pointed out in the course of a long discussion, by Sir Savile Crossley, M.P., one of the hon. secretaries of the Fund, Sir Chandos Leigh, and Mr. Cameron, that, as the Bill was originally drafted, the appointment of president was left to the nomination of the King. In the House of Lords, however, this provision was modified so as to secure that future presidents could only be appointed on the recommendation of the Lord Chancellor, the Prime Minister, and the Governor of the Bank of England, and that they were to hold office only during the pleasure of the Sovereign.

Sir Savile Crossley asked whether the committee could suggest any better method of selecting the president.

The Chairman said he was quite satisfied with the method of selection for future presidents, but the difficulty that was pressing on his mind was this. It might not always be possible to have a person who would enjoy the same measure of public confidence as the present President. There might not be such a person to be got at times, and, in these circumstances, was it wise to give all these powers to future presidents? It occurred to him whether these exceptional powers might not be maintained for the present president, but that the same powers should not be enjoyed by future presidents?

The same view was expressed by Mr. Beale and Mr. Crombie (of the committee), and Mr. Caldwell said that, now that the fund had grown to such an extent, it should be put on a wider basis, and there should be less of that personal control which was, perhaps, necessary at the inception of the fund.

Sir Savile Crossley said it was only right to say that the Prince of Wales had given an immense amount of time and work to the fund, and that it had been the effort of his Royal Highness to bring in, and to take into confidence, as far as possible, all those who were interested in the London hospitals and had shown a willingness to help in the matter.

The Chairman said the promoters of the Bill now knew the character of the committee's doubts on the subject; and they would adjourn the further consideration of the measure until their next meeting (Thursday, June 13, 1907).

